# Polychromatic flow cytometry in evaluating rheumatic disease patients

**DOI:** 10.1186/s13075-015-0561-1

**Published:** 2015-03-05

**Authors:** Chungwen Wei, Scott Jenks, Iñaki Sanz

**Affiliations:** Department of Medicine, Division of Rheumatology and Lowance Center for Human Immunology, Emory University, 615 Michael Street, Atlanta, GA 30322 USA

## Abstract

B cells are central players in multiple autoimmune rheumatic diseases as a result of the imbalance between pathogenic and protective B-cell functions, which are presumably mediated by distinct populations. Yet the functional role of different B-cell populations and the contribution of specific subsets to disease pathogenesis remain to be fully understood owing to a large extent to the use of pauci-color flow cytometry. Despite its limitations, this approach has been instrumental in providing a global picture of multiple B-cell abnormalities in multiple human rheumatic diseases, more prominently systemic lupus erythematosus, rheumatoid arthritis and Sjogren’s syndrome. Accordingly, these studies represent the focus of this review. In addition, we also discuss the added value of tapping into the potential of polychromatic flow cytometry to unravel a higher level of B-cell heterogeneity, provide a more nuanced view of B-cell abnormalities in disease and create the foundation for a precise understanding of functional division of labor among the different phenotypic subsets. State-of-the-art polychromatic flow cytometry and novel multidimensional analytical approaches hold tremendous promise for our understanding of disease pathogenesis, the generation of disease biomarkers, patient stratification and personalized therapeutic approaches.

## Introduction

B cells play a central role in the pathogenesis of autoimmune diseases through a combination of antibody-dependent and antibody-independent mechanisms. The latter include, among others, antigen presentation, T-cell regulation, cytokine production and organization of secondary and tertiary lymphoid tissue [1]. The protective or pathogenic outcome of B-cell-mediated conditions (whether in autoimmunity, transplantation, infection or vaccination) is most probably due to the imbalanced participation of separate B-cell subsets with regulatory and effector functions or by the subversion of function of a given subset.

This functional richness has been mainly studied in the mouse, but is also starting to unravel in humans. Indeed, while definitive functional studies are harder to perform with human B cells, the availability of many well-defined surface and intracellular markers, including better markers of B-cell memory, have set the stage for informative human studies. Yet our ability to adjudicate functional significance and pathogenic relevance to separate B-cell populations on the basis of surface phenotype has remained limited. A major impediment to this endeavor is that human B-cell subsets are currently defined by pauci-color flow cytometry protocols that are often limited to IgD, CD27, CD38 and CD24 staining to classify the major accepted populations (transitional, naïve, memory and plasmablast subsets). The expression of other informative markers, including differentiation and activation markers and homing receptors, in these subsets is typically assessed through the use of several parallel panels. The limited use of available markers not only fails to differentiate multiple populations within the conventional core subsets, but also could potentially lead to erroneous attribution of functional properties. Hence, we believe it is imperative that polychromatic flow cytometry (PFC) is incorporated to fully characterize human B cells within a consistent classification [2]. In this review, we present the current knowledge of human B-cell subsets and their analysis in rheumatic diseases using flow cytometry. We summarize the data available for the best studied diseases, and discuss the potential use of the B-cell phenotype profile in stratifying patients, prognosticating the disease progression and evaluating the effectiveness of treatments.

## Review

### Human B-cell populations

As extensively reviewed elsewhere [3,4], the customarily used IgD/CD27 scheme classifies human peripheral blood CD19^+^ B cells into four core subsets: naïve IgD^+^CD27^−^ B cells, unswitched memory (UM) IgD^+^CD27^+^ B cells, switched memory (SM) IgD^−^CD27^+^ B cells and double-negative (DN) IgD^−^CD27^−^ switched B cells (refer to Table 1 for definitions). Plasmablasts are a rare population in steady-state healthy subjects and can be better discriminated as CD27^++^CD38^++^ cells within the IgD^−^ fraction. It should be noted that, in addition to mature naïve B cells, the IgD^+^CD27^−^ compartment also harbors transitional B cells. Although the fraction of transitional B cells in this compartment is fairly small in healthy subjects, it can be quite prominent in patients with autoimmune diseases such as systemic lupus erythematosus (SLE) either in untreated disease [5] or after B-cell depletion therapy [6]. Transitional B cells have traditionally been identified as CD24^++^CD38^++^ cells, and they can be distinguished from naive B cells in the IgD^+^CD27^−^ compartment by their lack of expression of the ABCB1 transporter and the resulting retention of dyes such as Rhodamine 123 and MitoTracker Green [7].

**Table 1:** **Phenotype of human B-cell subsets in the periphery**

**B-cell subset**		**Phenotype** ^**a**^	**Function/properties** ^**b**^	**Perturbation**
Transitional cells	T1/T2	**IgD** ^**+**^ **CD27** ^**−**^MTG^+^CD24^++^CD38^++^CD10^+^IgM^+^	Developmental precursor	↓ in SLE
	T3	**IgD** ^**+**^ **CD27** ^**−**^MTG^+^CD24^+^CD38^+^CD10^−^IgM^+^	Developmental precursor	↑ in SLE
Naïve cells	Resting	**IgD** ^**+**^ **CD27** ^**−**^MTG^−^CD21^+^CD24^+/−^CD38^+/−^IgM^+^CD95^−^	Developmental precursor	↓ in SLE, ↑ in SSc
	Activated	**IgD** ^**+**^ **CD27** ^**−**^MTG^+^CD21^−^CD24^−^CD38^−^IgM^+^CD95^+^	Precursor of short-lived plasmablast and GC reaction	↑ in SLE, ↑in SSc^c^
	Anergic	**IgD** ^**+**^ **CD27** ^**−**^MTG^−^CD24^−^CD38^−^IgM^low/–^	Hyporesponsive. Maintenance of tolerance	↓ in SLE
Memory cells	Unswitched	IgM^+^ **IgD** ^**+**^ **CD27** ^**+**^CD1c^+^	Natural memory marginal zone equivalent	↓ in SLE, RA, pSS
	IgM-only	IgM^+^ **IgD** ^**−**^ **CD27** ^**+**^	Pre-switch memory. Early IgM memory. IgG memory precursor	↑ in SLE
	Switched			
	Resting	**IgD** ^**−**^ **CD27** ^**+**^CD21^+^CD95^−^IgG/A^+^	Protective anti-microbial memory?	↓ in SLE
	Activated	**IgD** ^**−**^ **CD27** ^**+**^CD21^−^CD95^+^IgG/A^+^	Pathogenic autoimmune memory?	↑ in SLE, ↓ in pSS
	Double-negative	**IgD** ^**−**^ **CD27** ^**−**^IgM/G/A^+^	Tissue based-memory. Exhausted memory?^d^	↑ in SLE
Antibody secreting cells	Pre-plasmablasts	**IgD** ^**−**^ **CD27** ^**+/−**^ **CD38** ^**++**^CD138^−^Ki67^+^	Antibody secretion	↑ in SLE
	Plasmablasts	**IgD** ^**−**^ **CD27** ^**++**^ **CD38** ^**++**^CD138^−^Ki67^+^	Antibody secretion	↑ in SLE, RA^e^
	Plasma cells	**IgD** ^**−**^ **CD27** ^**++**^ **CD38** ^**++**^CD138^+^Ki67^+^	Antibody secretion	↑ in SLE, RA^e^
Regulatory B cells	Bregs	CD24^hi^CD38^hi^	IL-10 production	Loss of function in SLE
		CD24^hi^CD27^+^		
		IgD^+^CD27^+^CD43^+^CD70^−^CD11b^+^		
9G4^+^ B cells	9G4^+^	9G4^+^	VH4-34 encoded autoreactive B cells	↑ in SLE
RP105^−^ B cells	RP105^−^	RP105^−^ (IgD^−^CD38^hi^CD138^dull^)	Resemble antibody secreting cells	↑ in SLE, pSS

^a^Markers in bold font indicate commonly defined core subsets. ^b^References for the indicated function/properties are incorporated throughout the text. ^c^Defined as CD19^hi^CD21^low^ B cells. ^d^Unlike the exhausted memory cells in HIV-infected and malaria-infected subjects, double-negative cells in SLE do not express FCRL4. ^e^In RA, the increase in antibody secreting cells is observed in synovial tissues. GC, germinal center; IL, interleukin; pSS, primary Sjogren’s syndrome; RA, rheumatoid arthritis; SLE, systemic lupus erythematosus; SSC, systemic sclerosis.

Substantial phenotypic heterogeneity has been recognized among human memory B cells (as defined by the expression of CD27), although their functional heterogeneity is less well understood [8]. Approximately one-half of all human CD27^+^ memory B cells have undergone isotype switch (IgG^+^ and IgA^+^), and the rest express surface IgM with or without the concomitant expression of surface IgD [8,9]. While the classical switched memory (SM) cells are generated from the germinal center reaction, IgD^+^IgM^+^CD27^+^ unswitched memory (UM) cells have been proposed to represent circulating marginal zone B cells, which are critical for protection against infections with encapsulated bacteria [10]. A variable fraction of CD27^+^ memory cells express only surface IgM (IgM-only memory) and may represent pre-SM cells that will eventually join the pool of isotype SM cells after participating in subsequent germinal center reactions [9,11].

Also well established is the existence of a subset of isotype switched B cells lacking expression of CD27, an antigen widely considered a universal marker of human memory cells [12,13]. These cells are comparable with conventional CD27^+^ SM cells in that they are class switched and somatically mutated and they experience much greater proliferative responses than naïve B cells after TLR9 stimulation through CpG DNA in the absence of simultaneous B-cell receptor engagement. IgD^−^CD27^−^ double-negative (DN) switched cells undergo substantial expansion in SLE patients, and the degree of expansion correlates well with disease activity [12]. Similar to CD27^+^ SM cells, the IgD^−^CD27^−^ DN compartment contains a fraction of IgM-only cells as well as class switched IgG and IgA cells [12]. Overall, the origin and role of IgD^−^CD27^−^ DN B cells remain to be understood although a derivation from initial germinal center reactions has been suggested in some studies [14]. DN B cells resemble a tissue-based memory population phenotypically, but they do not express the characteristic FcRL4 cell surface marker in the peripheral blood of both healthy and SLE subjects [12]. However, FcRL4 expression in these cells was observed in HIV-infected viremic individuals and those chronically infected with malaria [15,16]. It has been suggested that, at least in chronic infections such as HIV and malaria, DN cells may represent prematurely exhausted cells owing to the influence of FcRL4 and possibly other inhibitory receptors [15-17]. Nonetheless, other studies have suggested their active participation in the generation of anti-malaria antibodies [18].

The concept of effector and regulatory functions of B cells in cellular immune responses has received great attention in recent years. Hence, it is important to discuss the different phenotypes proposed for regulatory B cells (Bregs), a population with protective effects in autoimmune conditions [19] and whose preservation or enhancement should be an important consideration in the design of B-cell targeting therapies. Bregs suppress inflammation and autoimmunity through the production of cytokine interleukin (IL)-10. In the mouse, Breg function has been ascribed to different cell types including B1 cells [20], marginal zone B cells [21], B10 cells with a CD1d^hi^CD5^+^ phenotype [22] and transitional cells [23]. Similarly, Breg function has been proposed in the human for naïve B cells (IgD^+^CD27^−^) [24], transitional B cells (CD24^hi^CD38^hi^) [25], B10 cells (CD24^hi^CD27^+^) [26] and orchestrator B1 cells (B1orc) [27]. Mouse B cells of the B1 lineage have been known to be a rich source of IL-10 [20], but the identity of the human B1 counterpart remains elusive.

Recently, a population of human peripheral blood B cells with a CD20^+^IgD^+^CD27^+^CD43^+^CD70^−^ phenotype has been shown to exhibit the functional hallmarks of the mouse B1 cells [28], although its actual significance and magnitude remain to be further explored. Of note, the CD11b^+^ fraction of this human B1 population, termed B1orc, spontaneously secretes IL-10 and suppresses T-cell activation [27]. Mouse B10 cells, designated to represent splenic IL-10-producing CD1d^hi^CD5^+^ B cells, share some phenotypic markers with other IL-10-producing cells including B1 and marginal zone B cells [22]. Human B10 cells, on the other hand, are predominantly found within the CD24^hi^CD27^+^ compartment [26].

Given the diverse cell types capable of exerting regulatory function, there is no definitive cell surface marker(s) that can serve as a surrogate for IL-10 production. Furthermore, there might be subtle differences in the regulatory capacity among the different Breg subsets. For instance, suppression of CD4^+^ T-cell proinflammatory cytokine tumor necrosis factor alpha (TNFα) production by human transitional B cells is dependent on IL-10 [25]. In contrast, human B10 cells do not seem to regulate CD4^+^ T-cell TNFα expression, although they do suppress TNFα production by monocytes [26].

Other unique populations of B cells can be relevant for specific autoimmune diseases. For instance, a group of B cells recognized by an anti-human idiotype 9G4 antibody represents a highly informative experimental model to understand the breakdown of B-cell tolerance in SLE. In healthy subjects, effective tolerance ensures that 9G4 responses are restricted to acute infections with mycoplasma and Epstein–Barr virus, and that they do not persist in the long-lived IgG memory and plasma cell compartments [29]. In contrast, we have shown that 9G4^+^ B cells are substantially expanded in the SLE IgG memory B-cell compartment, and 9G4^+^ antibodies contribute disproportionally to circulating IgG levels owing to defective germinal center censoring [30,31]. Among other autoreactivities, 9G4^+^ antibodies have been shown to constitute a major species of anti-apoptotic cell antibodies in SLE serum [32]. CD19^hi^ memory B cells are enriched in anti-Sm B cells in SLE, and the degree of enrichment correlates with the level of serum anti-Sm antibodies as well as with adverse outcome and poor response to ritxumibab in small studies [33].

Another B-cell subset, which lacks the expression of RP105, is increased in the peripheral blood of patients with SLE, Sjogren’s syndrome and dermatomyositis [34]. Particularly in SLE, the increase in this B-cell population, which has been shown to produce anti-double-stranded DNA antibodies, appears to correlate with disease activity [34]. Several lines of evidence, including surface phenotype (CD20^−^CD38^hi^CD138^dull^) and spontaneous production of antibodies *in vitro*, suggest that these RP105^−^ B cells consist of antibody secreting cells (ASC) [34]. Hence, the observed increase of RP105^−^ B cells is consistent with the expansion of ASC in SLE patients with active disease (discussed in the next section).

### B-cell abnormalities in human autoimmune diseases

#### Systemic lupus erythematosus

Multiple alterations in the composition of the B-cell compartment have been reported in SLE, arguably the autoimmune disease with most florid and variable changes in B-cell homeostasis. B-cell lymphopenia was one of the initial observations in SLE patients [35] and subsequent flow cytometry studies have shown decreased absolute numbers of both CD27^+^ and CD27^−^ B cells [36,37]. Additionally, the proportion of IgD^+^CD27^+^ memory B cells is dramatically reduced in SLE patients [36]. Unlike other alterations seen in SLE B-cell homeostasis, the loss of UM B cells is found in almost all SLE patients regardless of disease activity.

ASC, defined as CD27^bright^ cells, are expanded in SLE patients with active disease [36,38-40]. Of interest, this subset contains both CD138^−^ cells as well as CD138^+^ cells, despite their universal expression of Ki-67. Thus, even mature circulating ASC in active SLE appear to represent newly generated plasmablasts. The expansion of ASC in SLE patients with active disease probably reflects increased activation and differentiation. In addition to ASC, several studies have found activated memory B cells in SLE patients, as indicated by their expression of the B-cell co-stimulatory molecules CD80 and CD86 and the death receptor CD95 [41,42]. This activation is not limited to memory B cells, as these molecules are also upregulated in IgD^+^CD27^−^ naïve B cells of SLE patients that have increased size, indicating *in vivo* activation [43]. More B cells in SLE patients express high levels of CD19 and these cells are enriched for anti-Smith autoreactivity and show several markers of activation, including low expression of the complement receptor CD21, high levels of CD86 and phosphorylation of B-cell receptor signaling molecules in the absence of stimulation [33,44].

An activated phenotype is also observed in the IgD^−^CD27^−^ DN population. As described above, this population is a minor subset in healthy individuals that is class switched and has undergone somatic hypermutation, but lacks the memory marker CD27 [45]. In SLE patients this population can be dramatically expanded, and both the parental subset and its activated CD95^+^CD21^−^ fraction correlate with disease activity [12,41]. The extent to which these cells are a result of naïve and memory B-cell activation or instead derive through a distinct differentiation pathway is an unresolved question of significance for our understanding of SLE pathogenesis.

Finally, multiple abnormalities of putative Breg populations have also been reported in SLE, including the decreased ability of Bregs (CD24^hi^CD38^hi^) to inhibit T-cell and macrophage activation, despite increased cell numbers in active SLE patients [25]. Of note, a subset of this population (CD1d^+^) that powerfully induces suppressive invariant natural killer T cells has also been reported to be deficient in SLE [46]. Interestingly, the recovery of this population appears to correlate well with favorable outcome following rituximab-induced B-cell depletion [46]. Substantial abnormalities have also been reported for B10 and B1 cells in SLE. Their actual functional significance remains to be ascertained, as both B10 cells and their precursors (pro-B10 cells) as well as the IL-10-producing orchestrator B1 cells are increased in SLE patients [26,47].

#### Rheumatoid arthritis

Alterations of B-cell subsets in rheumatoid arthritis (RA) are variable. One study found a higher proportion of IgD^−^CD27^+^ memory and decreased numbers of naive B cells [48], while a separate study of RA B-cell subsets in a large patient cohort found decreased numbers of IgD^−^CD27^+^ memory and this correlated with high disease activity [49]. This discrepancy is probably explained by differences in patient populations, treatment status and disease duration. With regards to the latter parameter, very early in RA disease patients already exhibit decreased numbers of IgD^+^CD27^+^ memory B cells prior to treatment [50]. The loss of this population, which is also depleted in SLE and primary Sjogren’s syndrome (pSS) [51], is thus probably not the result of treatment or chronic autoimmunity and occurs instead either prior to or very soon after disease onset.

Overall, the magnitude of changes in B-cell populations in the blood of RA patients is smaller than those observed in other systemic autoimmune diseases and the main locus of B-cell dysregulation in RA may be at the site of inflammation rather than the periphery. B-cell trafficking is altered in RA, as a decreased number of peripheral blood B cells express the B-cell follicle homing receptor CXCR5, but exhibit increased expression of CXCR3 which promotes migration to inflamed tissues [52]. While the infiltrate found in inflamed synovial tissue includes T cells, B cells and monocytes, the presence of large numbers of B cells, particularly CD38^+^ plasma cells, is characteristic of RA as compared with other types of arthritis [53]. Subsequent flow cytometry studies have found that many of these infiltrating B cells are CD27^+^ memory cells [54]. Histologically, in patients with active RA, synovial B cells are found in aggregates in close proximity to T cells and follicular dendritic cells [55]. Less commonly, ectopic lymphoid tissue that resembles secondary follicles is also observed. Aggregates and follicles are the site of ongoing proliferation, as they contain B cells positive for the nuclear antigen Ki-67 [56]. Plasma cells surround these aggregates and sequencing studies demonstrate clonal expansions and ongoing diversification through somatic hypermutation [57]. However, shared clones have also been found between blood and synovial B cells and between B cells from different joints [58,59], and synovial B cells are probably a mix of *in situ* generated clones and clones from distal locations that subsequently migrate in response to inflammation. Recently an additional proinflammatory role for synovial B cells has been found in the form of RANKL-expressing FCRL4^+^ memory B cells that express TNFα and resemble the tissue-based memory B cells found in the tonsil [60].

The prominence of tissue-based B cells in RA has important implications for treatment. One proposed model postulates, based on the relative ineffectiveness of B-cell depletion in tissue, that anti-CD20 treatment probably acts by cutting off the source of new immigrating memory B cells from the blood [61]. This results in a slow attrition of synovial B cells and the eventual collapse of the self-perpetuating inflammatory process. Treatments that hasten this collapse could improve the efficacy of B-cell depletion treatment in RA.

#### Sjogren’s syndrome

In contrast to SLE where both CD27^+^ and CD27^−^ B-cell numbers are reduced, patients with pSS have a very specific loss of CD27^+^ memory B cells [62,63]. This is an actual numerical loss rather than a change in proportions and affects both IgD^+^ and IgD^−^ memory B cells [51,64]. Sjogren’s syndrome, in particular, is in need of better diagnostics, as Sicca symptoms are common in the general population and early diagnosis and treatment can prevent permanent organ damage. The loss of memory B cells in pSS is consistent enough that this phenotype has been proposed as a diagnostic tool and loss of B-cell memory, as measured by an alternative flow schema based on CD38 and IgD expression, has been tested for this purpose [65]. These studies found that the loss of B-cell memory was of diagnostic value, but did not provide an improvement over present classification criteria.

We have also recently examined CD27^+^ B cells in pSS and Sicca patients [51]. We found that CD27^+^ cells, and in particular IgD^+^CD27^+^ memory cells, are greatly reduced not only in established pSS patients but also in a subset of Sicca patients. Of great interest, decreased numbers of UM cells correlated with serological indicators of autoimmunity both in Sicca subjects as well as in patients with pSS. Additionally, the residual IgD^+^CD27^+^ had an altered phenotype both by cell surface expression and gene transcription profiling. As in SLE, why this population is absent in pSS patients remains unexplored. The salivary glands of pSS patients have lymphocyte aggregates, and in a subset of patients these aggregates form germinal center-like structures. CD27^+^ memory B cells are a portion of these infiltrates and low numbers of CD27^+^ B cells in the blood may be the result of selective homing to the target tissue [64]. However, CD27^+^ memory B cells in ectopic germinal centers are uncommon and additional mechanisms may also be responsible the reduction of CD27^+^ B cells in pSS [66].

#### Systemic sclerosis

The B-cell phenotype of systemic sclerosis (SSc) patients has some parallels with other B-cell-mediated autoimmune diseases, but there are also some interesting differences. Like pSS patients, SSc patients have reduced numbers of CD27^+^ B cells; but unlike SLE patients, these patients are not B-cell lymphopenic [67]. Instead, the number of B cells in SSc patients is actually increased due to an expansion of CD27^−^ B cells. It is unclear what proportion of this expansion is due to IgD^+^ naive B cells as opposed to IgD^−^CD27^−^ DN B cells. Both CD27^+^ and CD27^−^ B-cell subsets in SSc patients expressed higher levels of CD19 [68]. CD19 is an important B-cell co-receptor that augments signaling and decreases the threshold for B-cell activation. Studies in the tight skin mouse model of SSc have demonstrated that a 20% increase in CD19, similar in magnitude to that seen in patients, resulted in both increased B-cell signaling and higher levels of SSc-specific anti-topoisomerase [69].

While multiple autoantibodies can be seen in patients with SSc, unlike SLE patients they do not have elevated numbers of circulating CD27^bright^ plasma cells. Recently, a potential autoantibody-independent role for B cells in SSc has been suggested by work showing that B cells increased expression of collagen by cultured SSc dermal fibroblasts [70]. This increase was enhanced by B-cell activating factor (BAFF) and anti-IgM treatment and was transforming growth factor beta dependent. The fact that anti-IgM modulated this effect implicates naive B cells (expanded at least in the blood of SSc), although the experiment did not rule out a role for IgD^+^CD27^+^ memory cells.

#### Commonalities and differences in B-cell abnormalities in rheumatic diseases

The loss of IgD^+^CD27^+^ UM cells is the strongest commonality in B-cell phenotype among rheumatic diseases because it is observed in SLE, pSS and RA. The underlying cause of this intriguing abnormality remains to be elucidated. It is possible that splenic dysfunction in patients may disrupt the anatomical sites necessary for the development and/or survival of UM B cells, as has been reported in patients with Crohn’s disease and celiac disease [71,72]. Alternatively, alterations in B-cell receptor signaling or other pathways may favor their differentiation into other cell fates at the expense of the IgD^+^CD27^+^ pathway [73]. The functional consequences of the loss of UM cells remains unknown, but may explain the increased risk of SLE and Sjogren’s syndrome patients for developing pneumococcal disease [74], as IgD^+^CD27^+^ B cells are important responders against encapsulated bacteria [75]. Marginal zone B cells in mice possess a fraction of IL-10-producing Bregs [21], and in humans these IgD^+^CD27^+^ memory cells may also have regulatory functions, which are ultimately compromised in autoimmune patients.

The large expansion of plasma cells observed in SLE patients is relatively specific to SLE, as plasma cell frequencies are not elevated in other rheumatic diseases when compared with healthy controls. As mentioned above, prominent plasma cell populations are found in tissues in both pSS and RA, and one potential explanation for this difference is that plasma cell differentiation is happening primarily in the target tissues in pSS and RA. Consistent with the increase in active B cells seen in SLE, autoimmunity for SLE may be driven more by continual recruitment of new cells and autoimmunity for RA and pSS by long-lived plasma cells or reactivated memory. This model suggests that treatments which disrupt naive B cells such as BAFF inhibition may be particularly effective in the treatment of SLE and treatments that inhibit trafficking or retention in target tissues may be promising approaches for treating RA and pSS.

### B-cell-targeted therapies

B cells play a critical role in the pathogenesis of autoimmune diseases, so B-cell-targeted therapies have become an attractive treatment modality. Depending on the mechanisms of action, B-cell-targeting agents can be categorized into those that directly kill most B cells and those that compromise the survival, differentiation and activation of B cells. The latter class of agents tends to target discreet B-cell subsets. The efficacy of these more selective B-cell targeting agents will thus depend on their effect on specific B-cell subsets and the contribution of the affected subsets to regulatory or pathogenic functions. A precise understanding of the phenotype and function of different B-cell subsets is therefore of the essence for a rational design of B-cell-targeted therapies.

Rituximab, a chimeric anti-CD20 monoclonal antibody, was the first B-cell-targeting biologic agent to receive US Food and Drug Administration approval for the treatment of autoimmune rheumatic diseases. Rituximab induces universal depletion of all B cells except those that lack the expression of CD20, such as pro-B cells and plasma cells. Nonetheless, small numbers of residual memory B cells as well as plasmablasts can be detected in the peripheral blood at the point of maximal depletion even in patients with effective B-cell depletion [76]. Studies in SLE patients treated with rituximab show that different patterns of B-cell reconstitution would emerge that correlate well with the clinical outcomes of the treatment. Short-term responders are characterized by rapid accumulation of memory B cells and plasmablasts [76,77], most probably resulting from the preferential homeostatic proliferation and expansion of these residual cells. On the contrary, long-term responders demonstrate a delayed memory B-cell recovery and a prolonged expansion of transitional B cells [6,77]. Given the success of treating SLE with rituximab in many open studies, the failure of two recent randomized, placebo-controlled trials (EXPLORER and LUNAR) to show added values from rituximab over conventional therapy was quite unexpected [78,79]. Aside from the other plausible explanations for the failure, the degree of initial B-cell depletion might have impacted the efficacy [80], as demonstrated by the recent studies in which a deeper B-cell depletion increases the efficacy of rituximab treatment in RA [81,82].

In contrast to direct killing of pan B cells by rituximab, other B-cell-targeted therapies induce quite different B-cell changes reflecting different mechanisms of action. For instance, belimumab, a monoclonal antibody that blocks BAFF binding to its receptors, preferentially inhibits the survival and hence decreases the numbers of transitional and activated naïve B cells [83,84]. The numbers of CD27^+^ SM cells and plasma cells are not affected, indicating these subsets are independent of BAFF for survival. However, a subset of IgD^−^CD27^−^ DN switched cells undergoes significant and sustained reduction [83]. Reflecting both the impact of sample size and duration of follow-up as well as the impact of different definitions and measurement of seemingly similar cell types, discordant results have been reported regarding the impact of belimumab on plasmablasts and other ASC [83,84]. Epratuzumab, another monoclonal antibody, exerts an agonistic effect on the inhibitor receptor CD22, further dampening B-cell activation. In contrast to rituximab, epratuzumab does not drastically deplete circulating B cells, but induces an average reduction of peripheral B cells by 30% mainly in the CD27^−^ compartment [85], which includes transitional cells, naïve cells as well as IgD^−^CD27^−^ switched cells. Attenuation of the B-cell receptor signaling pathway can also be mediated through the inhibition of tyrosine kinases such as Syk and Btk by small molecules that were developed initially to treat B-cell lymphomas [86]. Although the effects of these inhibitors on nonmalignant B cells are largely unknown, a recent study shows that short-term use of the Syk inhibitor fostamatinib in lymphoma patients impairs B-cell development at the transitional stage without affecting mature B-cell populations [87]. Even though two recent phase 3 clinical trials of fostamatinib in RA were a disappointment, other agents that target the B-cell receptor signaling pathway hold significant promises in treating autoimmune diseases [88].

### Polychromatic flow cytometry analysis of human B cells

**Figure 1:**
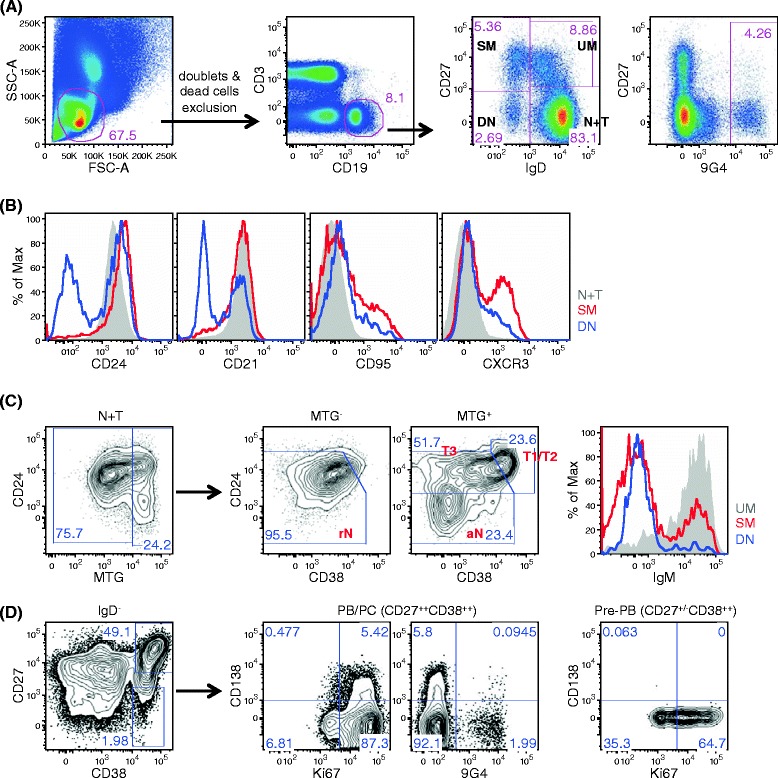
**Gating schemes for the analysis of human B cells. (A)** Cell aggregates and dead cells were further removed from the lymphocyte population, and the resulting live CD19^+^CD3^−^ cells were selected for analysis. The customarily used IgD/CD27 scheme classifies peripheral blood B cells into four core subsets: naïve and transitional (N + T) IgD^+^CD27^−^ B cells, unswitched memory (UM) IgD^+^CD27^+^ B cells, switched memory (SM) IgD^−^CD27^+^ B cells, and double-negative (DN) IgD^−^CD27^−^ B cells. Rightmost panel: autoreactive 9G4^+^ B cells concentrate within the naïve compartment. **(B)** With the additional memory panel specific markers, SM and DN cells both exhibit heterogeneous subpopulations. A great majority of DN cells downregulate the expression of CD24 and CD21, while CD95^+^ and CXCR3^+^ cells are more frequently observed in SM cells. **(C)** MitoTracker Green (MTG) in the transitional panel separates IgD^+^CD27^−^ N + T cells into MTG^−^ resting naïve cells (rN) and MTG^+^ fraction. The latter can be further subset into early (T1/T2) transitional B cells, late (T3) transitional B cells and activated naïve (aN) B cells based on the CD24/CD38 expression pattern. A sizable IgM-only memory cells can be identified in the SM subset as well as in the DN subset. **(D)** Plasma cell panel illustrates that IgD^−^CD27^++^CD38^++^ cells include CD138^−^ plasmablasts (PB) and CD138^+^ plasma cells (PC); both subsets are highly proliferative in the peripheral blood. The IgD^−^CD27^−/+^CD38^++^ region contains a CD24^−^ fraction that is also highly proliferative and is considered to be a pre-plasmablast subset (Pre-PB). 9G4^+^ plasmablasts are readily identified from systemic lupus erythematosus patients. FSC, forward scatter; SSC, side scatter.

Our knowledge of human B-cell subsets and of the perturbation of their homeostasis in disease could be exploited to apply B-cell profiling as a means to optimize disease diagnosis, prognosis and treatment. To achieve this goal, a comprehensive B-cell phenotyping is of the essence. Hence, we have developed several 12-color panels for in-depth characterization of memory cells, naïve/transitional cells and ASC [2-4] (Table 2). These panels share seven anchor markers, a B-cell lineage and two exclusion markers (CD19, CD3 and Live/Dead) as well as four developmental markers (IgD, CD27, CD38 and CD24), which enable the precise identification of the same core human B-cell subsets across panels (Figure 1A). Also common to all three panels is an anti-idiotype 9G4 antibody, which provides a useful measure of autoreactivity through the identification of B cells expressing autoantibodies encoded by the VH4-34 variable region gene [30]. Panel-specific markers then allow in-depth characterization of these core B-cell subsets and aid the identification of potentially novel subsets. The incorporation of CD21, CD95 and CXCR3 in the memory panel thus provides information regarding the activation status and homing potential of the memory B cells. The addition of MitoTracker Green in the transitional panel further segregates the late transitional (T3) cells from the resting naïve population. CD138 and Ki-67 in the plasma cell panel provide additional information on the subsets and proliferation status of plasmablasts/plasma cells (Figure 1).

**Table 2:** **Composition of the staining panels for human B-cell phenotyping**

	**Memory panel**		**Transitional panel**		**Plasma cell panel**	
**Fluorochrome**	**Specificity**	**Clone**	**Specificity**	**Clone**	**Specificity**	**Clone**
FITC	**IgD**	IA6-2	MTG	Free dye	Ki-67	B56
PE	CXCR3	1C6	**IgD**		CXCR3	1C6
PE-Alexa610	**CD24**	SN3	**CD24**		**CD24**	
PE-Cy5	CD21	B-ly4	IgM	G20-127	CXCR4	12G5
PerCP-Cy5.5	**CD38**	HIT2	**CD38**		**CD3**	
PE-Cy7	CD45/B220	RA3/6B2	CD23	EBVCS2	**CD38**	
Pacific Blue	**CD3**	SP34-2	**CD3**		**VH4-34**	
Pacific Orange	**Live/Dead**	Aqua	**Live/Dead**		**Live/Dead**	
Qdot605	**CD27**	CLB-27/1	**CD27**		**CD27**	
APC	CD95	DX2	CD10	HI10a	CD138	B-B4
Alexa680	**VH4-34**	9G4	**VH4-34**		**IgD**	
APC-Cy7	**CD19**	SJ25C1	**CD19**		**CD19**	

The seven anchor markers and VH4-34 encoded heavy chain are in bold font. Information on the detecting antibody clones for these eight molecules is omitted from the transitional and plasma cell panels, as they are the same as those indicated under the memory panel regardless of the fluorochrome conjugates. For detection in the Red B channel, antibodies used are biotinylated and detected by streptavidin-Alexa680. Note that in the plasma cell panel, both Ki-67 and 9G4 are intracellular staining. APC, allophycocyanin; Aqua, LIVE/DEAD fixable aqua dead cell stain; Cy, cyanine; FITC, fluorescein isothiocyanate; PE, R-phycoerythrin; PerCP, peridinin-chlorophyll protein; Qdot, quantum dot.

Increasingly complex high-dimensional PFC data create new challenges for data mining and interpretation. Just as challenging is the difficulty in the level of standardization required for large datasets and multicenter studies typical of large clinical trials [89]. These challenges are being proactively addressed by many groups, often on a collaborative basis, to develop various clustering algorithms that can identify discrete cell populations based on simultaneous assessment of multiple parameters, and hold significant promise for the automated analysis of PFC data [90,91]. To overcome the time-consuming and variable nature of manual gating, a normalization algorithm has been developed that, when integrated into the manual template gating procedure, is capable of mitigating the sample-to-sample variation and allows for high-throughput processing of large PFC datasets [89]. Likewise, recently developed software called AutoGate is promising to become an automated tool for processing and analyzing PFC data [92].

**Figure 2:**
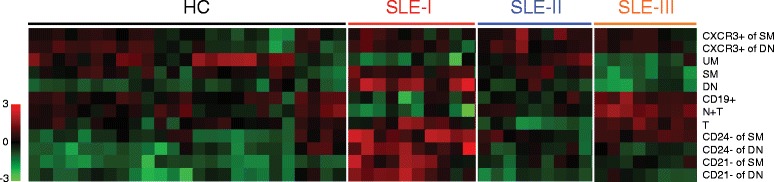
**Unsupervised clustering analysis of B-cell profiles segregates lupus patients into distinct groups.** Flow cytometry data from 25 healthy controls (HC) and 137 systemic lupus erythematosus (SLE) patients were clustered independently by B-cell phenotypic profiles using Matlab (MathWorks, Natick MA, USA). Clustering was based on Euclidean distance and complete linkage using a reduced feature set to avoid correlated cell subsets based on the gating strategy. Subset frequencies (in rows) were logit-transformed and each cell subset was standardized to its mean and standard deviation of all 162 samples (in columns) prior to clustering. This approach segregated lupus patients into three distinct clusters, and representative lupus patients from each cluster were shown. Preliminary analysis indicates that SLE-I cluster is enriched for patients with high Systemic Lupus Erythematosus Disease Activity Index and high serum interferon alpha activity, in contrast to SLE-II cluster whose B-cell profile resembles that of healthy controls (manuscript in preparation). Note that the B-cell profiles among the healthy controls are relatively heterogeneous. Subset frequencies are the percentages of total B cells, unless indicated otherwise. CD19^+^ frequencies are percentages of lymphocytes. DN, double negative; N + T, IgD^+^CD27^−^ fraction that contains both naïve and transitional cells; SM, switched memory; T, CD24^++^CD38^++^ transitional B cells; UM, unswitched memory.

Traditionally, the frequency (or absolute number) of each B-cell subset derived from flow cytometry analysis is presented independent of that of other subsets, largely as part of univariate analysis. Perturbation of B-cell homeostasis in a disease state is often described separately for each affected subset as discussed in the previous section. However, univariate approaches on individual subsets fail to reveal how collections of subsets and their relative distributions might contribute to patient groupings. Thus, we have applied a global B-cell profiling approach, in which all of the B-cell subset data are considered simultaneously to obtain a system-wide view of B-cell populations [4,51,93]. In this manner, patient-specific complex B-cell fingerprints are generated that can be directly compared with the profile of other patients. An unsupervised hierarchical clustering analysis can then divide patients into groups based on their B-cell profiles (Figure 2), and stringent correlations of B-cell fingerprints with clinical, immunological and other emergent features can be identified. Our results from a large multicenter study [93] (and manuscript in preparation) provide a proof of concept that, when combined with other informative clinical parameters, B-cell profiling offers a systems biology approach to identifying potential biomarkers for the diagnosis, prognosis and treatment monitoring of lupus disease.

## Conclusions

Flow cytometry has emerged as a powerful tool for B-cell immunophenotyping through the use of increasing number of markers, as well as the incorporation of intracellular staining to interrogate the functional properties such as the production of cytokines and phosphorylation of key signaling molecules [2,87,94-96]. Two recent innovations have further extended the utility of flow cytometry in important ways. Mass cytometry (cytometry by time of flight) uses time-of-flight mass spectrometry to measure heavy metal isotopes conjugated to antibodies rather than fluorescence. Because there is little signal overlap between isotopes, many more parameters (>40) can be measured at the same time [97]. These additional parameters may be particularly useful in signaling pathway studies using phosphoprotein-specific antibodies, as multiple pathways can be integrated and combined with cell surface phenotype. Recently this approach has been used to characterize innate cell responses to influenza vaccine [98]. Equivalent studies of B cells could extend already known alterations in autoimmune B-cell receptor signaling [95] by simultaneously analyzing Toll-like receptor and cytokine signaling to understand how these pathways intersect and are dysregulated in rheumatic disease.

Imaging cytometry combines the throughput of flow cytometry with fluorescent microscopy imaging capabilities. Rather than providing only quantification, these images provide information on molecular localization and cell morphology while still maintaining the high throughput and multiple parameter advantages of flow cytometry [99]. Molecular localization is necessary to understand many important biological processes. As an example, image cytometry was used to quantify autophagosomes in human and mouse B cells, demonstrating that autophagy was increased in SLE and was required for plasmablast development [100]. Cell morphology data from imaging cytometry were also used to establish the importance of cell polarization and asymmetric cell division in B-cell antigen processing, a phenomenon that has important implications for B-cell differentiation and functional diversity [101].

The advances in PFC technology provide unprecedented opportunities to carry out a large number of measurements at the single-cell level in a high-throughput fashion. This approach should provide a high-level definition of the complexity of human B cells and of the multiple changes that characterize rheumatic diseases and their response to treatment in general, and B-cell-targeting agents in particular. In turn, this level of definition should bear tremendous implications for the way we identify, characterize and treat these diseases. B-cell profiles may serve as biomarkers to estimate risk of disease progression and to initiate early treatment that might halt disease progression or improve long-term outcome. Moreover, careful definition of B-cell phenotype by PFC will enable the elucidation of the functional properties of the different populations and of the molecular roadmaps responsible for their abnormal behavior in disease, thereby leading to the identification of new therapeutic targets.

Moving forward, our understanding of B cells in human autoimmunity will be greatly enhanced by the consistent use of a homogeneous nomenclature and multi-color staining protocols with shared phenotypic markers. The impact of these studies will be maximized by the ongoing development of automated, multidimensional analytical programs and shared public databases accessible to the research community. Finally, it will be essential to perform larger longitudinal studies that incorporate detailed clinical information, to compare diverse autoimmune conditions under the same experimental and analytical parameters and to analyze autoimmune patients before and after therapeutic intervention with B-cell-targeting interventions.

## Abbreviations

ASC: antibody secreting cells
BAFF: B-cell activating factor
Breg: regulatory B cell
DN: double-negative
IL: interleukin
PFC: polychromatic flow cytometry
pSS: primary Sjogren’s syndrome
RA: rheumatoid arthritis
SLE: systemic lupus erythematosus
SM: switched memory
SSc: systemic sclerosis
TNFα: tumor necrosis factor alpha
UM: unswitched memory

## References

[1] Manjarrez-Orduno N, Quach TD, Sanz I (2009). B cells and immunological tolerance. J Invest Dermatol.

[2] Wei C, Jung J, Sanz I (2011). OMIP-003: phenotypic analysis of human memory B cells. Cytometry A.

[3] Kaminski D, Wei C, Rosenberg A, Lee FE-H, Sanz I, Perl A (2012). Multiparameter flow cytometry and bioanalytics for B cell profiling in systemic lupus erythematosus. Autoimmunity.

[4] Kaminski DA, Wei C, Qian Y, Rosenberg AF, Sanz I (2012). Advances in human B cell phenotypic profiling. Front Immunol.

[5] Sims GP, Ettinger R, Shirota Y, Yarboro CH, Illei GG, Lipsky PE (2005). Identification and characterization of circulating human transitional B cells. Blood.

[6] Palanichamy A, Barnard J, Zheng B, Owen T, Quach T, Wei C (2009). Novel human transitional B cell populations revealed by B cell depletion therapy. J Immunol.

[7] Wirths S, Lanzavecchia A (2005). ABCB1 transporter discriminates human resting naive B cells from cycling transitional and memory B cells. Eur J Immunol.

[8] Klein U, Rajewsky K, Kuppers R (1998). Human immunoglobulin (Ig)M + IgD+ peripheral blood B cells expressing the CD27 cell surface antigen carry somatically mutated variable region genes: CD27 as a general marker for somatically mutated (memory) B cells. J Exp Med.

[9] Klein U, Kuppers R, Rajewsky K (1997). Evidence for a large compartment of IgM-expressing memory B cells in humans. Blood.

[10] Weller S, Braun MC, Tan BK, Rosenwald A, Cordier C, Conley ME (2004). Human blood IgM ‘memory’ B cells are circulating splenic marginal zone B cells harboring a prediversified immunoglobulin repertoire. Blood.

[11] Dogan I, Bertocci B, Vilmont V, Delbos F, Megret J, Storck S (2009). Multiple layers of B cell memory with different effector functions. Nat Immunol.

[12] Wei C, Anolik J, Cappione A, Zheng B, Pugh-Bernard A, Brooks J (2007). A new population of cells lacking expression of CD27 represents a notable component of the B cell memory compartment in systemic lupus erythematosus. J Immunol.

[13] Sanz I, Wei C, Lee FE-H, Anolik J (2008). Phenotypic and functional heterogeneity of human memory B cells. Semin Immunol.

[14] Berkowska MA, Driessen GJA, Bikos V, Grosserichter-Wagener C, Stamatopoulos K, Cerutti A (2011). Human memory B cells originate from three distinct germinal center-dependent and -independent maturation pathways. Blood.

[15] Moir S, Ho J, Malaspina A, Wang W, DiPoto AC, O’Shea MA (2008). Evidence for HIV-associated B cell exhaustion in a dysfunctional memory B cell compartment in HIV-infected viremic individuals. J Exp Med.

[16] Weiss GE, Crompton PD, Li S, Walsh LA, Moir S, Traore B (2009). Atypical memory B cells are greatly expanded in individuals living in a malaria-endemic area. J Immunol.

[17] Sohn HW, Krueger PD, Davis RS, Pierce SK (2011). FcRL4 acts as an adaptive to innate molecular switch dampening BCR signaling and enhancing TLR signaling. Blood.

[18] Muellenbeck MF, Ueberheide B, Amulic B, Epp A, Fenyo D, Busse CE (2013). Atypical and classical memory B cells produce Plasmodium falciparum neutralizing antibodies. J Exp Med.

[19] Fillatreau S, Sweenie CH, McGeachy MJ, Gray D, Anderton SM (2002). B cells regulate autoimmunity by provision of IL-10. Nat Immunol.

[20] O’Garra A, Chang R, Go N, Hastings R, Haughton G, Howard M (1992). Ly-1 B (B-1) cells are the main source of B cell-derived interleukin 10. Eur J Immunol.

[21] Lenert P, Brummel R, Field EH, Ashman RF (2005). TLR-9 activation of marginal zone B cells in lupus mice regulates immunity through increased IL-10 production. J Clin Immunol.

[22] Yanaba K, Bouaziz JD, Haas KM, Poe JC, Fujimoto M, Tedder TF (2008). A regulatory B cell subset with a unique CD1dhiCD5+ phenotype controls T cell-dependent inflammatory responses. Immunity.

[23] Evans JG, Chavez-Rueda KA, Eddaoudi A, Meyer-Bahlburg A, Rawlings DJ, Ehrenstein MR (2007). Novel suppressive function of transitional 2 B cells in experimental arthritis. J Immunol.

[24] Duddy M, Niino M, Adatia F, Hebert S, Freedman M, Atkins H (2007). Distinct effector cytokine profiles of memory and naive human B cell subsets and implication in multiple sclerosis. J Immunol.

[25] Blair PA, Norena LY, Flores-Borja F, Rawlings DJ, Isenberg DA, Ehrenstein MR (2010). CD19(+)CD24(hi)CD38(hi) B cells exhibit regulatory capacity in healthy individuals but are functionally impaired in systemic lupus erythematosus patients. Immunity.

[26] Iwata Y, Matsushita T, Horikawa M, Dilillo DJ, Yanaba K, Venturi GM (2011). Characterization of a rare IL-10-competent B-cell subset in humans that parallels mouse regulatory B10 cells. Blood.

[27] Griffin DO, Rothstein TL (2012). Human ‘orchestrator’ CD11b(+) B1 cells spontaneously secrete interleukin-10 and regulate T-cell activity. Mol Med.

[28] Griffin DO, Holodick NE, Rothstein TL (2008). Human B1 cells in umbilical cord and adult peripheral blood express the novel phenotype CD20 + CD27 + CD43 + CD70–. J Exp Med.

[29] Milner EC, Anolik J, Cappione A, Sanz I (2005). Human innate B cells: a link between host defense and autoimmunity?. Springer Semin Immunopathol.

[30] Pugh-Bernard AE, Silverman GJ, Cappione AJ, Villano ME, Ryan DH, Insel RA (2001). Regulation of inherently autoreactive VH4-34 B cells in the maintenance of human B cell tolerance. J Clin Invest.

[31] Cappione A, Anolik JH, Pugh-Bernard A, Barnard J, Dutcher P, Silverman G (2005). Germinal center exclusion of autoreactive B cells is defective in human systemic lupus erythematosus. J Clin Invest.

[32] Jenks SA, Palmer EM, Marin EY, Hartson L, Chida AS, Richardson C (2013). 9G4+ Autoantibodies are an important source of apoptotic cell reactivity associated with high levels of disease activity in systemic lupus erythematosus. Arthritis Rheum.

[33] Nicholas MW, Dooley MA, Hogan SL, Anolik J, Looney J, Sanz I (2008). A novel subset of memory B cells is enriched in autoreactivity and correlates with adverse outcomes in SLE. Clin Immunol.

[34] Koarada S, Tada Y (2012). RP105-negative B cells in systemic lupus erythematosus. Clin Dev Immunol.

[35] Scheinberg MA, Cathcart ES (1974). B cell and T cell lymphopenia in systemic lupus erythematosus. Cell Immunol.

[36] Odendahl M, Jacobi A, Hansen A, Feist E, Hiepe F, Burmester GR (2000). Disturbed peripheral B lymphocyte homeostasis in systemic lupus erythematosus. J Immunol.

[37] Potter KN, Mockridge CI, Rahman A, Buchan S, Hamblin T, Davidson B (2002). Disturbances in peripheral blood B cell subpopulations in autoimmune patients. Lupus.

[38] Arce E, Jackson DG, Gill MA, Bennett LB, Banchereau J, Pascual V (2001). Increased frequency of pre-germinal center B cells and plasma cell precursors in the blood of children with systemic lupus erythematosus. J Immunol.

[39] Jacobi AM, Mei H, Hoyer BF, Mumtaz IM, Thiele K, Radbruch A (2010). HLA-DRhigh/CD27high plasmablasts indicate active disease in patients with systemic lupus erythematosus. Ann Rheum Dis.

[40] Minowa K, Amano H, Nakano S, Ando S, Watanabe T, Nakiri Y (2011). Elevated serum level of circulating syndecan-1 (CD138) in active systemic lupus erythematosus. Autoimmunity.

[41] Jacobi AM, Reiter K, Mackay M, Aranow C, Hiepe F, Radbruch A (2008). Activated memory B cell subsets correlate with disease activity in systemic lupus erythematosus: delineation by expression of CD27, IgD, and CD95. Arthritis Rheum.

[42] Dolff S, Wilde B, Patschan S, Durig J, Specker C, Philipp T (2007). Peripheral circulating activated B-cell populations are associated with nephritis and disease activity in patients with systemic lupus erythematosus. Scand J Immunol.

[43] Chang N-H, McKenzie T, Bonventi G, Landolt-Marticorena C, Fortin PR, Gladman D (2008). Expanded population of activated antigen-engaged cells within the naive B cell compartment of patients with systemic lupus erythematosus. J Immunol.

[44] Wehr C, Eibel H, Masilamani M, Illges H, Schlesier M, Peter H-H (2004). A new CD21low B cell population in the peripheral blood of patients with SLE. Clin Immunol.

[45] Fecteau JF, Cote G, Neron S (2006). A new memory CD27-IgG+ B cell population in peripheral blood expressing VH genes with low frequency of somatic mutation. J Immunol.

[46] Bosma A, Abdel-Gadir A, Isenberg DA, Jury EC, Mauri C (2012). Lipid-antigen presentation by CD1d(+) B cells is essential for the maintenance of invariant natural killer T cells. Immunity.

[47] Griffin DO, Rothstein TL (2011). A small CD11b + human B1 cell subpopulation stimulates T cells and is expanded in lupus. J Exp Med.

[48] Fekete A, Soos L, Szekanecz Z, Szabo Z, Szodoray P, Barath S (2007). Disturbances in B- and T-cell homeostasis in rheumatoid arthritis: suggested relationships with antigen-driven immune responses. J Autoimmun.

[49] Sellam J, Rouanet S, Hendel-Chavez H, Abbed K, Sibilia J, Tebib J (2011). Blood memory B cells are disturbed and predict the response to rituximab in patients with rheumatoid arthritis. Arthritis Rheum.

[50] Moura RA, Weinmann P, Pereira PA, Caetano-Lopes J, Canhao H, Sousa E (2010). Alterations on peripheral blood B-cell subpopulations in very early arthritis patients. Rheumatology(Oxford).

[51] Roberts MEP, Kaminski D, Jenks SA, Maguire C, Ching K, Burbelo PD (2014). Primary Sjögren’s syndrome is characterized by distinct phenotypic and transcriptional profiles of IgD+ unswitched memory cells. Arthritis Rheumatol.

[52] Henneken M, Dorner T, Burmester GR, Berek C (2005). Differential expression of chemokine receptors on peripheral blood B cells from patients with rheumatoid arthritis and systemic lupus erythematosus. Arthritis Res Ther.

[53] Kraan MC, Haringman JJ, Post WJ, Versendaal J, Breedveld FC, Tak PP (1999). Immunohistological analysis of synovial tissue for differential diagnosis in early arthritis. Rheumatology (Oxford).

[54] Nanki T, Takada K, Komano Y, Morio T, Kanegane H, Nakajima A (2009). Chemokine receptor expression and functional effects of chemokines on B cells: implication in the pathogenesis of rheumatoid arthritis. Arthritis Res Ther.

[55] Kim HJ, Krenn V, Steinhauser G, Berek C (1999). Plasma cell development in synovial germinal centers in patients with rheumatoid and reactive arthritis. J Immunol.

[56] Krenn V, Schalhorn N, Greiner A, Molitoris R, Konig A, Gohlke F (1996). Immunohistochemical analysis of proliferating and antigen-presenting cells in rheumatoid synovial tissue. Rheumatol Int.

[57] Scheel T, Gursche A, Zacher J, Haupl T, Berek C (2011). V-region gene analysis of locally defined synovial B and plasma cells reveals selected B cell expansion and accumulation of plasma cell clones in rheumatoid arthritis. Arthritis Rheum.

[58] Doorenspleet ME, Klarenbeek PL, de Hair MJ, van Schaik BD, Esveldt RE, van Kampen AH (2014). Rheumatoid arthritis synovial tissue harbours dominant B-cell and plasma-cell clones associated with autoreactivity. Ann Rheum Dis.

[59] Voswinkel J, Weisgerber K, Pfreundschuh M, Gause A (1999). The B lymphocyte in rheumatoid arthritis: recirculation of B lymphocytes between different joints and blood. Autoimmunity.

[60] Yeo L, Lom H, Juarez M, Snow M, Buckley CD, Filer A, et al. Expression of FcRL4 defines a pro-inflammatory, RANKL-producing B cell subset in rheumatoid arthritis. Ann Rheum Dis. 2014 Jan 15. doi:10.1136/annrheumdis-2013-204116. [Epub ahead of print] 10.1136/annrheumdis-2013-204116 PMC4392201 24431391

[61] Kavanaugh A, Rosengren S, Lee SJ, Hammaker D, Firestein GS, Kalunian K (2008). Assessment of rituximab’s immunomodulatory synovial effects (ARISE trial). 1: Clinical and synovial biomarker results. Ann Rheum Dis.

[62] Bohnhorst JO, Thoen JE, Natvig JB, Thompson KM (2001). Significantly depressed percentage of CD27+ (memory) B cells among peripheral blood B cells in patients with primary Sjogren’s syndrome. Scand J Immunol.

[63] Bohnhorst JO, Bjorgan MB, Thoen JE, Jonsson R, Natvig JB, Thompson KM (2002). Abnormal B cell differentiation in primary Sjogren’s syndrome results in a depressed percentage of circulating memory B cells and elevated levels of soluble CD27 that correlate with Serum IgG concentration. Clin Immunol.

[64] Hansen A, Odendahl M, Reiter K, Jacobi AM, Feist E, Scholze J (2002). Diminished peripheral blood memory B cells and accumulation of memory B cells in the salivary glands of patients with Sjogren’s syndrome. Arthritis Rheum.

[65] Cornec D, Saraux A, Pers JO, Jousse-Joulin S, Marhadour T, Roguedas-Contios AM (2014). Diagnostic accuracy of blood B-cell subset profiling and autoimmunity markers in Sjogren’s syndrome. Arthritis Res Ther.

[66] Aqrawi LA, Brokstad KA, Jakobsen K, Jonsson R, Skarstein K (2012). Low number of memory B cells in the salivary glands of patients with primary Sjogren’s syndrome. Autoimmunity.

[67] Sato S, Fujimoto M, Hasegawa M, Takehara K (2004). Altered blood B lymphocyte homeostasis in systemic sclerosis: expanded naive B cells and diminished but activated memory B cells. Arthritis Rheum.

[68] Sato S, Hasegawa M, Fujimoto M, Tedder TF, Takehara K (2000). Quantitative genetic variation in CD19 expression correlates with autoimmunity. J Immunol.

[69] Sato S, Fujimoto M, Hasegawa M, Takehara K, Tedder TF (2004). Altered B lymphocyte function induces systemic autoimmunity in systemic sclerosis. Mol Immunol.

[70] Francois A, Chatelus E, Wachsmann D, Sibilia J, Bahram S, Alsaleh G (2013). B lymphocytes and B-cell activating factor promote collagen and profibrotic markers expression by dermal fibroblasts in systemic sclerosis. Arthritis Res Ther.

[71] Di Sabatino A, Rosado MM, Miele L, Capolunghi F, Cazzola P, Biancheri P (2007). Impairment of splenic IgM-memory but not switched-memory B cells in a patient with celiac disease and splenic atrophy. J Allergy Clin Immunol.

[72] Di Sabatino A, Rosado MM, Ciccocioppo R, Cazzola P, Morera R, Corazza GR (2005). Depletion of immunoglobulin M memory B cells is associated with splenic hypofunction in inflammatory bowel disease. Am J Gastroenterol.

[73] Pillai S, Cariappa A (2009). The follicular versus marginal zone B lymphocyte cell fate decision. Nat Rev Immunol.

[74] Wotton CJ, Goldacre MJ (2012). Risk of invasive pneumococcal disease in people admitted to hospital with selected immune-mediated diseases: record linkage cohort analyses. J Epidemiol Commun Health.

[75] Carsetti R, Pantosti A, Quinti I (2006). Impairment of the antipolysaccharide response in splenectomized patients is due to the lack of immunoglobulin M memory B cells. J Infect Dis.

[76] Anolik JH, Barnard J, Cappione A, Pugh-Bernard AE, Felgar RE, Looney RJ (2004). Rituximab improves peripheral B cell abnormalities in human systemic lupus erythematosus. Arthritis Rheum.

[77] Anolik JH, Barnard J, Owen T, Zheng B, Kemshetti S, Looney RJ (2007). Delayed memory B cell recovery in peripheral blood and lymphoid tissue in systemic lupus erythematosus after B cell depletion therapy. Arthritis Rheum.

[78] Merrill JT, Neuwelt CM, Wallace DJ, Shanahan JC, Latinis KM, Oates JC (2010). Efficacy and safety of rituximab in moderately-to-severely active systemic lupus erythematosus: the randomized, double-blind, phase II/III systemic lupus erythematosus evaluation of rituximab trial. Arthritis Rheum.

[79] Rovin BH, Furie R, Latinis K, Looney RJ, Fervenza FC, Sanchez-Guerrero J (2012). Efficacy and safety of rituximab in patients with active proliferative lupus nephritis: the lupus nephritis assessment with rituximab study. Arthritis Rheum.

[80] Sanz I, Lee FE-H (2010). B cells as therapeutic targets in SLE. Nat Rev Rheumatol.

[81] Vital EM, Rawstron AC, Dass S, Henshaw K, Madden J, Emery P (2011). Reduced-dose rituximab in rheumatoid arthritis: efficacy depends on degree of B cell depletion. Arthritis Rheum.

[82] Vital EM, Dass S, Buch MH, Rawstron AC, Emery P. An extra dose of rituximab improves clinical response in rheumatoid arthritis patients with initial incomplete B cell depletion: a randomised controlled trial. Ann Rheum Dis. 2014 Jan 17. doi:10.1136/annrheumdis-2013-204544. [Epub ahead of print] 10.1136/annrheumdis-2013-204544 24443001

[83] Jacobi AM, Huang W, Wang T, Freimuth W, Sanz I, Furie R (2010). Effect of long-term belimumab treatment on b cells in systemic lupus erythematosus: extension of a phase II, double-blind, placebo-controlled, dose-ranging study. Arthritis Rheum.

[84] Stohl W, Hiepe F, Latinis KM, Thomas M, Scheinberg MA, Clarke A (2012). Belimumab reduces autoantibodies, normalizes low complement levels, and reduces select B cell populations in patients with systemic lupus erythematosus. Arthritis Rheum.

[85] Jacobi AM, Goldenberg DM, Hiepe F, Radbruch A, Burmester GR, Dörner T (2008). Differential effects of epratuzumab on peripheral blood B cells of patients with systemic lupus erythematosus versus normal controls. Ann Rheum Dis.

[86] Woyach JA, Johnson AJ, Byrd JC (2012). The B-cell receptor signaling pathway as a therapeutic target in CLL. Blood.

[87] Barr PM, Wei C, Roger J, Schaefer-Cutillo J, Kelly JL, Rosenberg AF (2012). Syk inhibition with fostamatinib leads to transitional B lymphocyte depletion. Clin Immunol.

[88] Avalos AM, Meyer-Wentrup F, Ploegh HL (2014). B-cell receptor signaling in lymphoid malignancies and autoimmunity. Adv Immunol.

[89] Finak G, Jiang W, Krouse K, Wei C, Sanz I, Phippard D (2014). High-throughput flow cytometry data normalization for clinical trials. Cytometry A.

[90] Qian Y, Wei C, Eun-Hyung Lee F, Campbell J, Halliley J, Lee JA (2010). Elucidation of seventeen human peripheral blood B-cell subsets and quantification of the tetanus response using a density-based method for the automated identification of cell populations in multidimensional flow cytometry data. Cytometry B Clin Cytom.

[91] Aghaeepour N, Finak G, Hoos H, Mosmann TR, Brinkman R, Gottardo R (2013). Critical assessment of automated flow cytometry data analysis techniques. Nat Methods.

[92] Meehan S, Walther G, Moore W, Orlova D, Meehan C, Parks D (2014). AutoGate: automating analysis of flow cytometry data. Immunol Res.

[93] Jung J, Biear J, Huang Y, Neary B, Marin E, Hossler J (2011). The comprehensive B cell profiling analysis of a multicenter SLE cohort. Arthritis Rheum.

[94] Biancotto A, Fuchs JC, Williams A, Dagur PK, McCoy JP (2011). High dimensional flow cytometry for comprehensive leukocyte immunophenotyping (CLIP) in translational research. J Immunol Methods.

[95] Jenks SA, Sanz I (2009). Altered B cell receptor signaling in human systemic lupus erythematosus. Autoimmun Rev.

[96] Taher TE, Parikh K, Flores-Borja F, Mletzko S, Isenberg DA, Peppelenbosch MP (2010). Protein phosphorylation and kinome profiling reveal altered regulation of multiple signaling pathways in B lymphocytes from patients with systemic lupus erythematosus. Arthritis Rheum.

[97] Bendall SC, Nolan GP, Roederer M, Chattopadhyay PK (2012). A deep profiler’s guide to cytometry. Trends Immunol.

[98] O’Gorman WE, Huang H, Wei YL, Davis KL, Leipold MD, Bendall SC (2014). The split virus influenza vaccine rapidly activates immune cells through Fcgamma receptors. Vaccine.

[99] Basiji DA, Ortyn WE, Liang L, Venkatachalam V, Morrissey P (2007). Cellular image analysis and imaging by flow cytometry. Clin Lab Med.

[100] Clarke AJ, Ellinghaus U, Cortini A, Stranks A, Simon AK, Botto M, et al. Autophagy is activated in systemic lupus erythematosus and required for plasmablast development. Ann Rheum Dis. 2014 Jan 13. doi:10.1136/annrheumdis-2013-204343. [Epub ahead of print] 10.1136/annrheumdis-2013-204343 PMC4152192 24419333

[101] Thaunat O, Granja AG, Barral P, Filby A, Montaner B, Collinson L (2012). Asymmetric segregation of polarized antigen on B cell division shapes presentation capacity. Science.

